# Vertebral plate and ligament composite laminoplasty in spinal cord tumor surgery: Analysis of 94 patients

**DOI:** 10.1515/tnsci-2021-0007

**Published:** 2021-01-20

**Authors:** Xu Hao, Wang Lin

**Affiliations:** Department of Neurosurgery, The First Affiliated Hospital of University of Science and Technology of China, Hefei, Anhui, China

## Abstract

**Objectives:**

The aim of this study was to evaluate the value and long-term effect of laminectomy or laminoplasty in spinal cord tumor surgery.

**Patients and methods:**

Patients with spinal cord tumor treated in Department of Neurosurgery from January 2016 to October 2019 were included in this study. Posterior median approach tumor resection was preceded in 94 cases. Vertebral plate and ligament composite replant (laminoplasty group) was proceeded in 34 cases, and vertebral plate resection (laminectomy group) was proceeded in 60 cases. All patients were followed up and neurological function imagings were conducted 1 week, 3 months, and 6 months postsurgery to evaluate the surgical efficiency and spinal stability.

**Results:**

Total resection was achieved in 84 patients (89.0%); subtotal resection was achieved in 10 patients (11%). There was no significant difference between thelaminectomy group and laminoplasty group in terms of operative time, surgical site, infection rate, cerebrospinal fluid (CSF) infection, CSF leak, and length of hospitalization (*P* > 0.05). The incidence of postoperative spinal deformity was 15.0% in the laminectomy group and 11.7% in the laminoplasty group (*P* > 0.05). Laminoplasty vs laminectomy was associated with a similar risk of progressive deformity. However, for the cervical patients, there is significant difference (*P* < 0.05) in the spinal deformity. For the patients with incision vertebral segments >3, there is no significant difference in the spinal deformity (*P* > 0.05). Bone fusion was achieved in 7 (20%) patients in the laminoplasty group. Laminoplasty vs laminectomy was associated with a similar risk of progressive deformity.

**Conclusion:**

Vertebral plate and ligament composite replant is a simple and practical method in spinal cord tumor surgery. Neither every case got bone fusion nor positive results turned out in survival analysis, but it is still valuable in reducing spinal deformity, especially in cervical vertebra spinal cord tumor surgery.

## 1 Introduction

Intradural spinal tumor is the most common spinal tumor, accounting for 4.3–10.4% in central nervous system tumor [1]. While posterior median approach is the most common method, both laminectomy and laminoplasty were well used in the procedure. Previous research indicated that the progressive spinal deformity would deteriorate the long-term function in many cases. The deformity rate is 7–20%, according to different studies [2,3,4,5], and for children the deformity rate could be 20–100% [5–8].

The technique of cervical laminoplasty was developed to decompress the spinal canal in patients with compression caused by ossification of the posterior longitudinal ligament or cervical spondylosis [9].

Theoretically, laminoplasty is considered as a practical method to reconstruct normal anatomical structure, preserve spinal stability, and prevent kyphosis compared to laminectomy. But there is no convincing clinical evidence to be recommended widely [10]. The aim of this study is to explore the difference between laminoplasty and laminectomy in short-term prognosis, neurofunction recovery, and the incidence and time of spinal deformity occurrence.

## 2 Patients and methods

### 2.1 Patient population

Patients with spinal cord tumor treated in Department of Neurosurgery from January 2016 to October 2019 were included in this study. Inclusion criteria were patients with spinal cord tumor including intramedullar tumors (such as ependymoma and astrocytoma); extramedullar, intradural tumors (such as neurinoma and meningioma); and epidural tumors (chordomas, teratomas, hemangiomas, and carcinomas metastases).

Exclusion criteria were metastasis tumor, any other cases in which decompression is required (such as chiari malformation, spinal stenosis, and ligamentum flavum calcification), and patients younger than 14 years. All patients from January 2016 to January 2018 were selected for laminectomy; patients presenting from February 2018 to October 2019 were selected for either laminectomy or laminoplasty on the basis of surgeons’ preference. Patients underwent preoperative and postoperative magnetic resonance imaging (MRI) in all cases. All patients were followed up with serial MRI and targeted CT to assess sagittal alignment 1 week, 3 months, and 6 months postsurgery, then annually. The neurological functions were also assessed accordingly.

Functional status was graded according to a modified McCormick scale both preoperatively and at the follow-up [11]. Preoperative radiographs were assessed for spinal deformity which was defined as progression of scoliotic or kyphotic curves by at least 10°. The onset time of progressive deformity was recorded.


**Informed consent:** Informed consent has been obtained from all individuals included in this study.
**Ethical approval:** The research related to human use has been complied with all the relevant national regulations, institutional policies, and in accordance the tenets of the Helsinki Declaration and has been approved by the authors’ institutional review board or equivalent committee.

### 2.2 Surgical techniques

**Figure 1:**
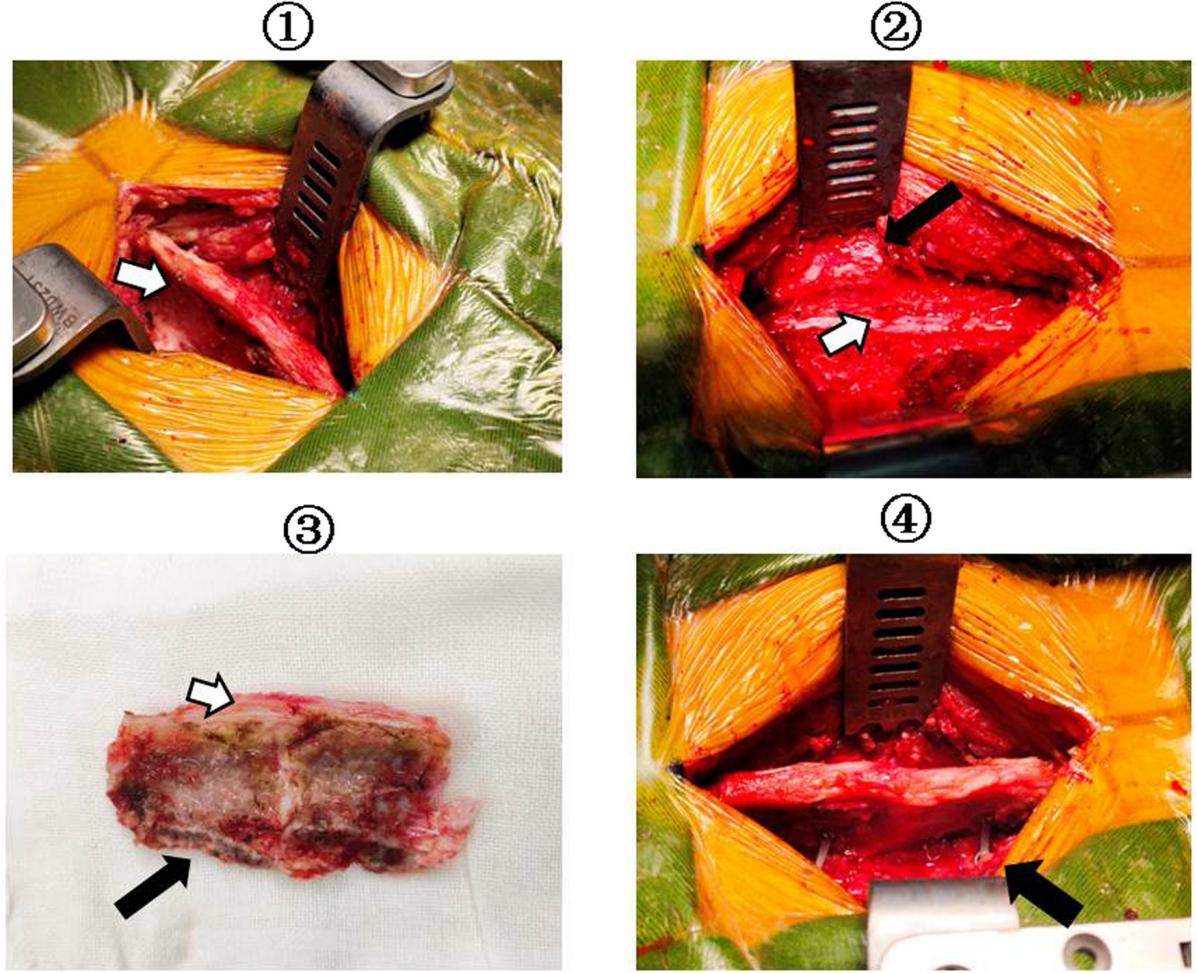
The technique of vertebral plate and ligament composite laminoplasty. ① Exposure of the spinous process; ⇨: the complete supraspinous ligament. ② Using abrasion drill and milling cutter complete resection the vertebral plate and ligament composite; ➡: bone fracture surface; ⇨: exposure of spinal dura mater. ③ Vertebral plate and ligament composite ➡: bone fracture surface; ⇨: vertebral plate and ligament composite. ④ Screws and connectors were used for fixation of the spinous process ligament complex; ➡: restore ligament integrity.

**Figure 2:**
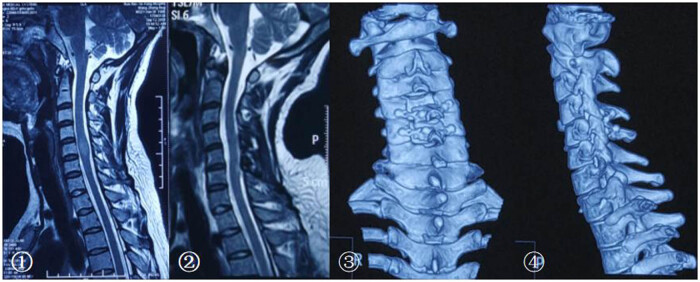
The MRI and CT imaging pre and postsurgery. ① T2 MRI before surgery. Postoperative pathology confirmed that it is an ependymocytoma. ② T2 MRI in 1 year postsurgery, there is no recurrence of the tumor and no progressive spinal deformity. ③ and ④ CT three-dimensional reconstruction showed that vertebral plate and ligament complex were in a suitable location.

The decision to perform laminoplasty was based on surgeons’ preference. For laminoplasty, only medial facet joint exposure is performed by subperiosteal paraspinal muscle dissection. An effort was made to preserve the facet joint capsules in all cases. Abrasion drill and milling cutter were used to resect the spinous process ligament complex completely. The spinous processes of the planned laminoplasty segment were left intact to preserve the interspinous ligaments and ligamentum flavum [12]. Vertebral plate and ligament composite was installed, then screws and connectors were used for fixation (Figure 1). Patients who underwent laminectomy had laminae removal above the entire length of the tumor. Sensory-evoked and motor-evoked potentials were used in all cases. All patients including cervical, thoracic, and lumbar vertebrae received external fixation postsurgery for at least 3 months (Figure 2).

### 2.3 Statistical analysis

For intergroup comparison, the Student *t* test was used for parametric data and the Mann–Whitney *U* test for nonparametric data. Percentages were compared via *χ* ^2^ tests. The absolute incidence of postoperative deformity was compared by *χ* ^2^ tests. The incidence of deformity was analyzed by the Kaplan–Meier method and then compared between laminoplasty and laminectomy groups via the log-rank test.

## 3 Results

### 3.1 Patient population

A total of 94 patients underwent surgical resection of an intradural spinal tumor during the reviewed time period. Of them, 60 (64%) underwent laminectomy and 34 (36%) underwent laminoplasty. The tumor location included cervical vertebra in 36 cases, thoracic vertebra in 21 cases, and lumbar vertebra in 37 cases. Total resection was achieved in 84 patients (89.0%); subtotal resection was achieved in 10 patients (11%). The tumor location included cervical vertebra in 36 cases, thoracic vertebra in 21 cases, and lumbar vertebra in 37 cases. Total resection was achieved in 84 patients (89.0%); subtotal resection was achieved in 10 patients (11%) (Table 1).

**Table 1:** Patient population

	All patients	Laminectomy (60)	Laminoplasty (34)
Mean age	45	47	44
M/F	58/36	38/22	20/14
Duration of symptoms (months)	3	4 (1–17)	3 (1–16)
Levels ≤ 2	57	33	24
Levels > 3	37	19	14
Cervical	36	21	13
Thoracic	21	14	7
Lumber	37	25	12

Pathology included ependymoma in 11 (12%), low-grade astrocytoma in 9 (10%), malignant astrocytoma in 2 (2%), cavernoma in 3 (3%), schwannoma in 31 (33%), angiolipoma in 11 (12%), meningioma in 11 (12%), neurofibroma in 7 (7%), epidermoid cyst in 5 (5%), enterogenous cysts in 3 (3%), and epidural simple cyst in 2 (2%).

The patients were followed up for a total of 17 months. Surgical site infection occurred in one patient (1%), whereas cerebrospinal fluid (CSF) infection occurred in five patients (6%). The incisional CSF leakage occurred in eight patients (9%). Mean length of hospitalization was 11 ± 4 days. There was no significant difference between the two methods in terms of operative time, surgical site, infection rate, CSF infection, incisional CSF leak, and length of hospitalization. In terms of neurological function, the McCormick score change also showed no difference between the two groups (*P* > 0.05) (Table 2). In two cases of laminoplasty, screw loose and slip off were detected in the follow-ups, but the patients showed no symptoms at that time. Intensive follow-ups were conducted accordingly.

**Table 2:** Laminoplasty vs laminectomy short-term prognosis

	All patients	Laminectomy	Laminoplasty	*P* value
Operative time (min)	135	124	141	0.382
Surgical site infection	1	0	1	
CSF infection	5 (5.32%)	3 (5.00%)	2 (5.88%)	0.987
Incisional CSF leakage	8	4	1	0.742
Length of hospitalization	11	11	11	0.997
McCormick score change	1.53	1.58	1.50	0.864

Thirteen (13.9%) patients developed progressive radiographic deformity, most of them (11) occurred in 1 year postsurgery. Nine patients developed progressive cervical deformity, two patients in lumbar, and two in thoracic vertebra, respectively.

Then, we subdivided the patients into two subgroups, including the cervical patient group and the group of patients with incision vertebral segments >3. We analyzed the deformity rate in the two groups in 12 months postsurgery. For the cervical patients, there is significant difference (*P* < 0.05) in the spinal deformity. For the patients with incision vertebral segments >3, there is no significant difference in the spinal deformity (*P* > 0.05) (Table 3).

**Table 3:** Laminoplasty vs laminectomy spinal stability in 12-month follow-up

All cases	Total	Laminectomy	Laminoplasty	*P* value
	94	60	34	
Deformity cases	13	9	4	0.6624
Cervical patients	36	21	15	
Deformity cases	9	8	1	0.0387
Levels > 3	33	19	14	
Deformity cases	9	6	3	0.5176

**Figure 3:**
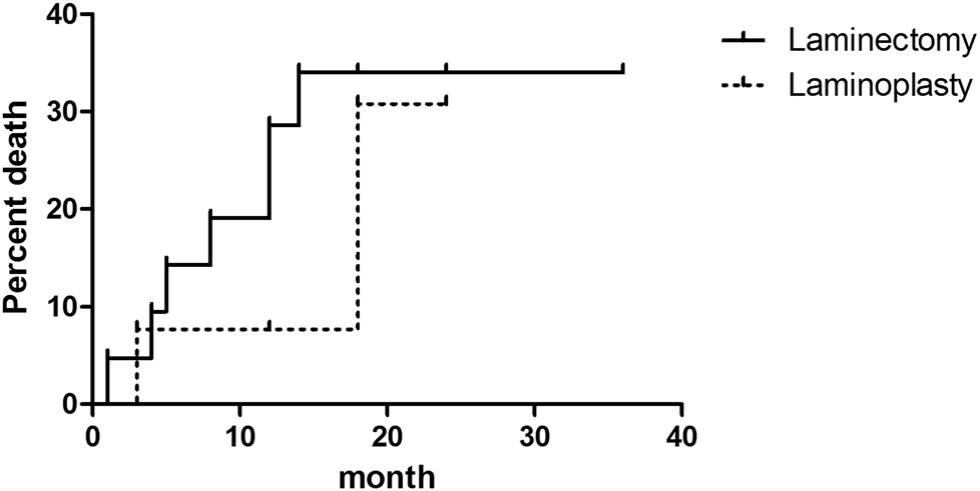
Kaplan–Meier curve of the incidence of progressive deformity after surgery of laminoplasty vs laminectomy in the cervical patient group.

**Figure 4:**
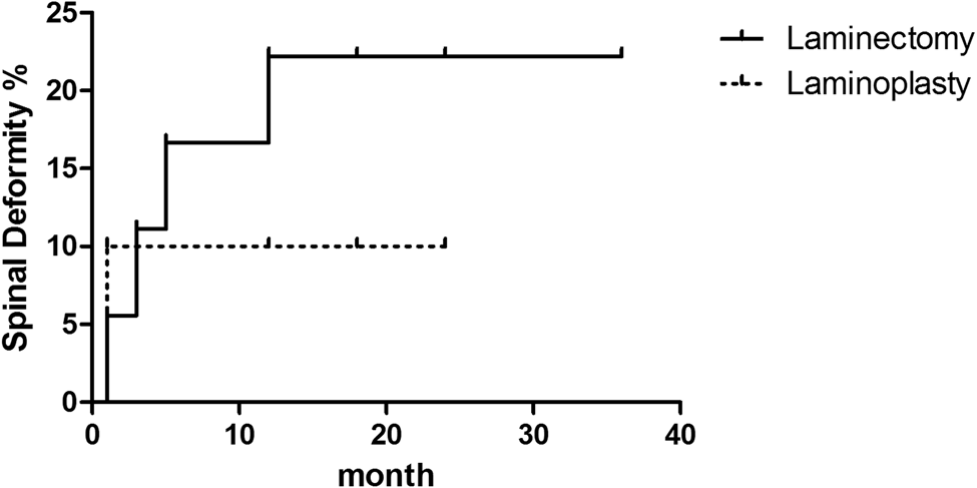
Kaplan–Meier curve of the incidence of progressive deformity after surgery of laminoplasty vs laminectomy in the group of patients with incision vertebral segments >3.

Subsequently, the incidence of progressive deformity after surgery was analyzed via the Kaplan–Meier method. In the cervical patient group, laminoplasty vs laminectomy was associated with a similar risk of progressive deformity (relative risk, 1.871; 95% CI, 0.4720 to 7.415, *P* = 0.3726) (Figure 3). In the group of patients with incision vertebral segments >3, laminoplasty vs laminectomy was associated with a similar risk of progressive deformity (relative risk, 2.018; 95% CI, 0.3201 to 12.72, *P* = 0.4549) (Figure 4).

## 4 Discussion

In this study, we assessed whether introducing laminoplasty into our practice would influence the incidence of postoperative short-term prognosis, neuro-functional recovery, and the spinal deformity occurrence following intradural tumor resection.

Laminectomy has long been considered as the traditional approach for intradural tumor resection because it is well established, creating a relatively wide exposure of the spinal cord, and can easily be extended in sagittal directions.

Adults undergoing laminectomy for intradural tumor resection developed postoperative progressive spinal deformity in 7–20% of all cases. Laminoplasty has therefore been advocated to avoid such complications because the posterior elements of the spinal cord are replaced [13,14]. This replacement is presumed to leave the posterior element intact, theoretically stabilizing the spine and preventing instability.

According to our research, there is no significant difference between the two methods in terms of complication rate and short-term prognosis. Vertebral plate and ligament composite reduction cannot reduce CSF leakage, which is different from previous studies [10]. Although screw loose and slip off were found in two cases of laminoplasty and the surgery time may be prolonged to some extent, it will not deteriorate the prognosis and can be avoided with more proficient surgical techniques.

Previous research indicate that in the occurrence of instability important factors included destroying of critical segments, such as C2, C7 [5,15]; the surgery incision more than three segments [16,17] and zygapophysis excision [17,18].

Due to a greater degree of cervical vertebra motion compared with thoracolumbar vertebra, coupled with the overuse of smart phones leading to a prolonged time of cervical anteflexion, we believed that cervical surgery is more likely to lead to spinal deformity and more attention should be paid to those patients. In terms of stability of the spine, the chi-square test in laminoplasty showed some advantages in cervical patients during 1-year follow-up. Even though there is no significant difference in subsequent survival analysis curve, we still believe that laminoplasty could be helpful for the spinal stability. At the same time, we also found that persistently wearing a neck brace for at least 3 months could reduce the risk of cervical spinal deformity.

For patients whose intraoperative diagnosis considered malignant tumor, we routinely performed laminectomy. However, we also found that for benign tumors, there were five patients who needed secondary surgery in this study. We also found that laminoplasty can reduce soft tissue hyperplasia and scar formation, restore anatomical layers, and make secondary surgery safer.

In addition, hemilaminectomy can better reduce the biomechanical damage, but on the other hand, hemilaminectomy will increase the risk of spinal cord injury because of the limited operating field [19]. Internal fixation, however, can better guarantee the stability of the spine, but at the same time also brought some unique complications and disadvantages. For example, screwing the pedicle could damage the vessel and nerve; fixation could destroy the original spine flexion, rotation, and other physiological functions; and pathological changes of segmental line could accelerate adjacent segment degeneration [20].

This study has some limitations because bias can be introduced in a retrospective review that does not have randomized, prospectively matched groups. Second, bias can also be caused by different surgery techniques. Despite the use of Kaplan–Meier methods to adjust for varying follow-up, our lack of long-term follow-up disallows any conclusions on long-term deformity is also a major limitation of this study.

## 5 Conclusion

Vertebral plate and ligament composite replant is a simple and practical method in spinal cord tumor surgery. Considering both strengths and weaknesses, it is still valuable to conduct in spinal cord tumor surgery especially in cervical vertebra surgery.
